# Supplementary material for: Interpretable Spatial Gradient Analysis for Spatial Transcriptomics Data
Source: bioRxiv. 2024 Mar 21:2024.03.19.585725. Preprint. [Version 1] doi: 10.1101/2024.03.19.585725 (PMC10983986; doi:10.1101/2024.03.19.585725)
Supplement: Supplement 4 [file NIHPP2024.03.19.585725v1-supplement-4.pdf]

bioRxiv preprint doi: https://doi.org/10.1101/2024.03.19.585725; this version posted March 21, 2024. The copyright holder for this preprint (which was not certified by peer review) is the author/funder, who has granted bioRxiv a license to display the preprint in perpetuity. It is made available under aCC-BY 4.0 International license.

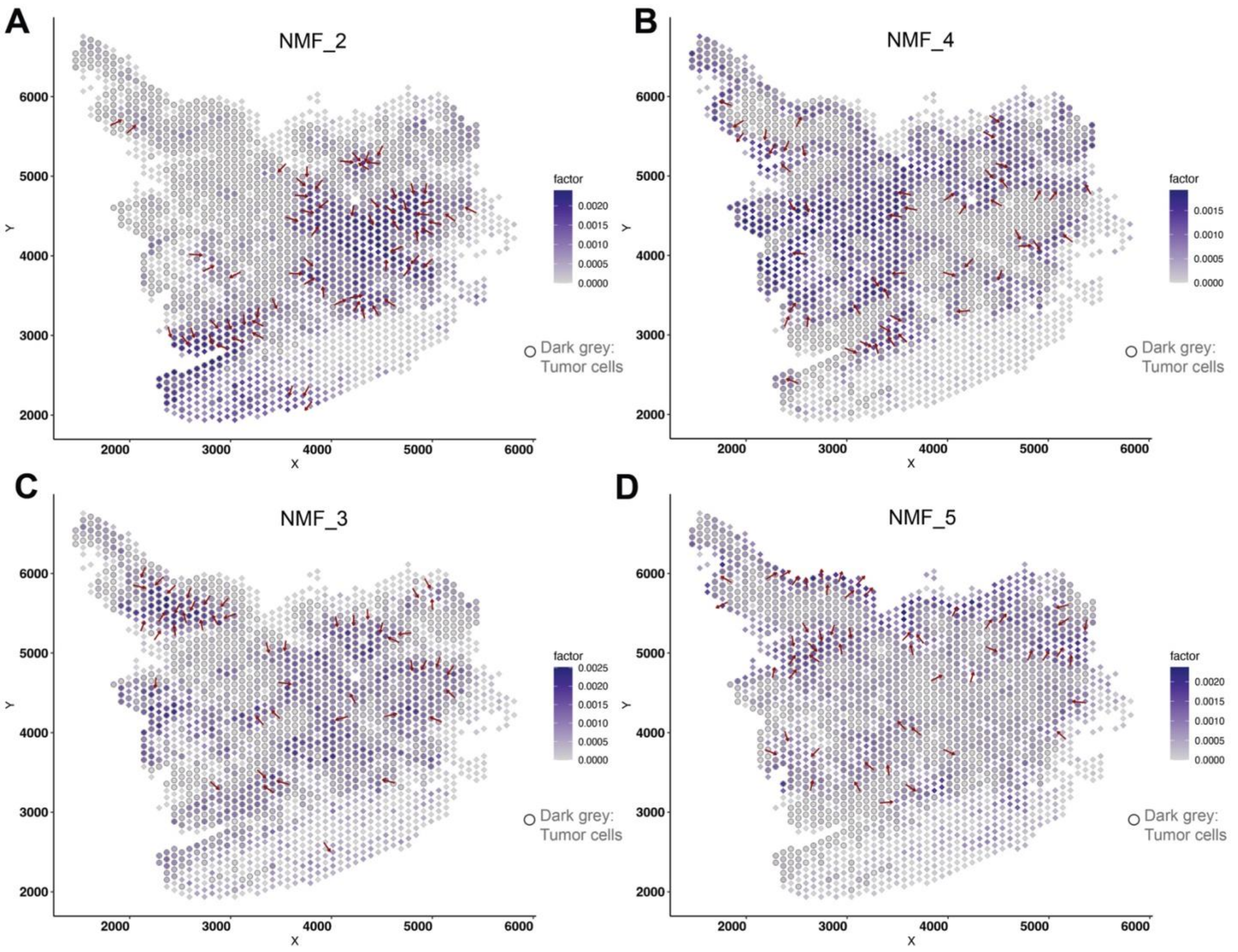

Supplementary Figure 1

A-D. Demonstration of the gradient direction and original cell loadings of NMF_2 (A), NMF_4 (B), NMF_3 (C), and NMF_5 (D) on the spatial map. The overlaying dark grey circles represent data spots characterized as tumor region

bioRxiv preprint doi: https://doi.org/10.1101/2024.03.19.585725; this version posted March 21, 2024. The copyright holder for this preprint (which was not certified by peer review) is the author/funder, who has granted bioRxiv a license to display the preprint in perpetuity. It is made available under aCC-BY 4.0 International license.

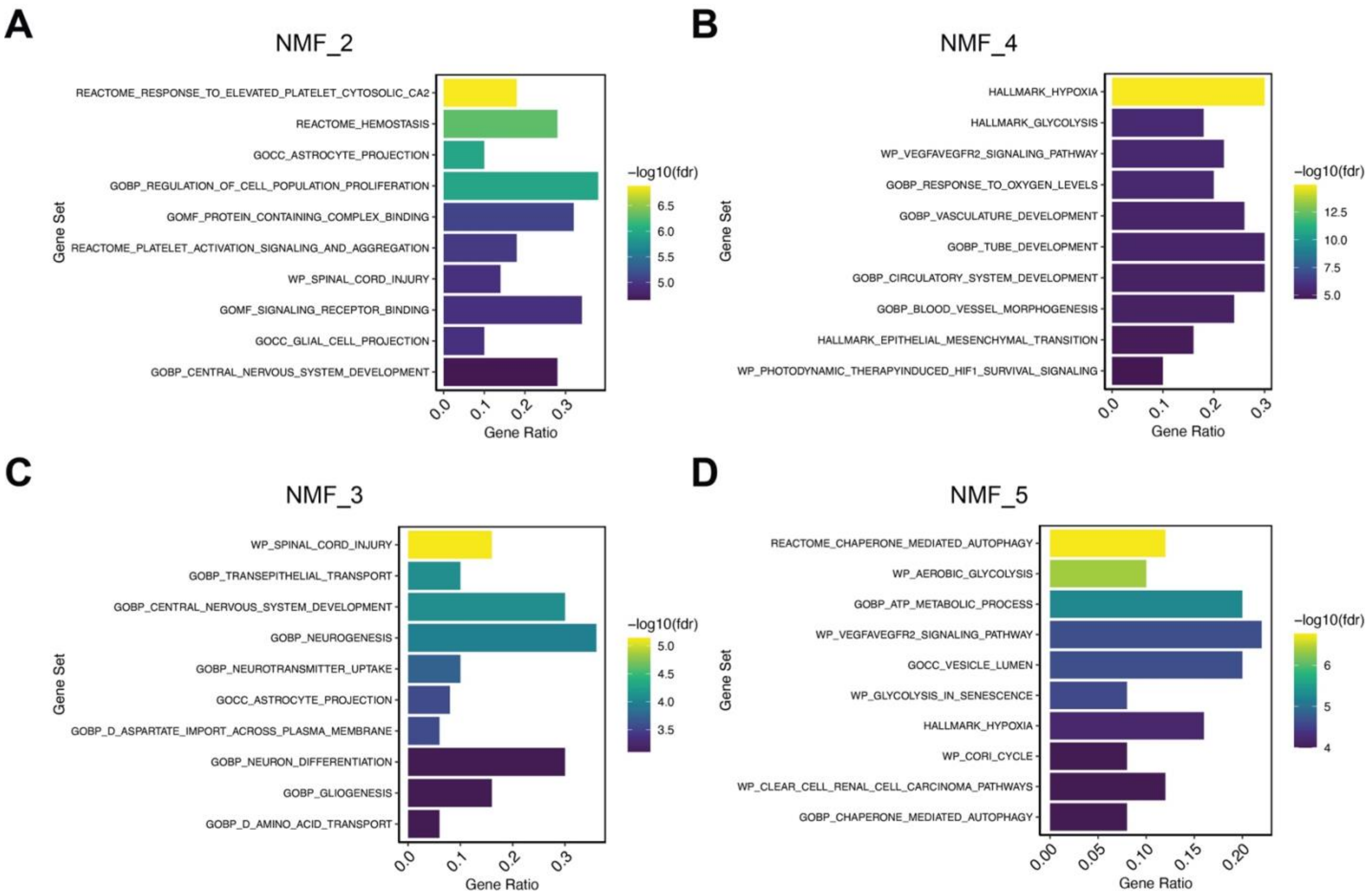

Supplementary Figure 2

A-D. Functional enrichment of top genes in NMF_2 (A), NMF_4 (B), NMF_3 (C), and NMF_5 (D). Bar-plots showed the ratio of pathway genes found in input gene sets (top 50 genes in each NMF program) and were colored by the adjusted p-value (false discovery rate, FDR) of hypergeometric test.

bioRxiv preprint doi: https://doi.org/10.1101/2024.03.19.585725; this version posted March 21, 2024. The copyright holder for this preprint (which was not certified by peer review) is the author/funder, who has granted bioRxiv a license to display the preprint in perpetuity. It is made available under aCC-BY 4.0 International license.

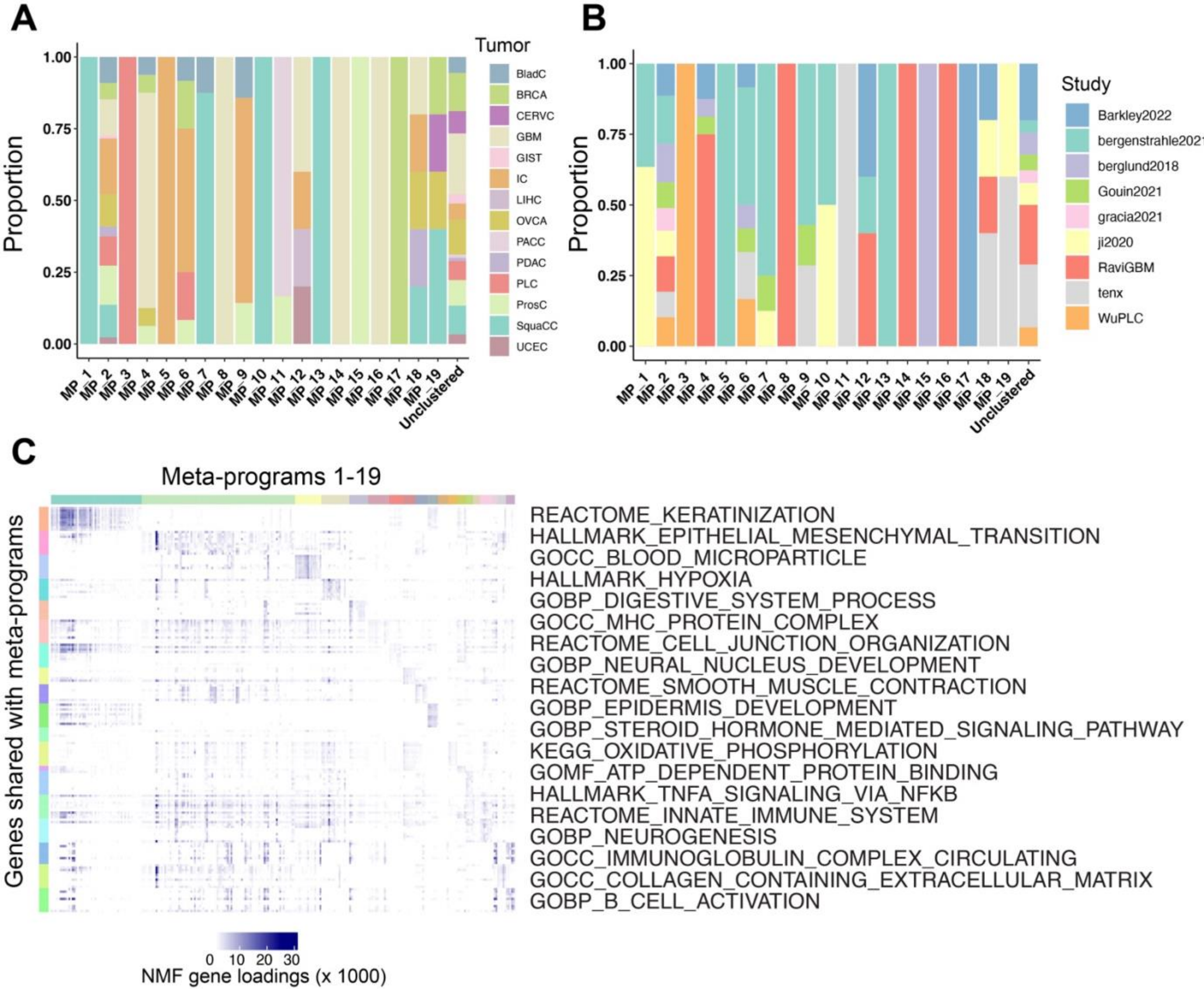

Supplementary Figure 3. Information of meta-program composition and functional annotations.

A-B. Proportion of originated tumor types and study of NMF programs in each meta-program.

C. Loadings of the assigned functional gene set members in each NMF program grouped by meta-program.

bioRxiv preprint doi: https://doi.org/10.1101/2024.03.19.585725; this version posted March 21, 2024. The copyright holder for this preprint (which was not certified by peer review) is the author/funder, who has granted bioRxiv a license to display the preprint in perpetuity. It is made available under aCC-BY 4.0 International license.

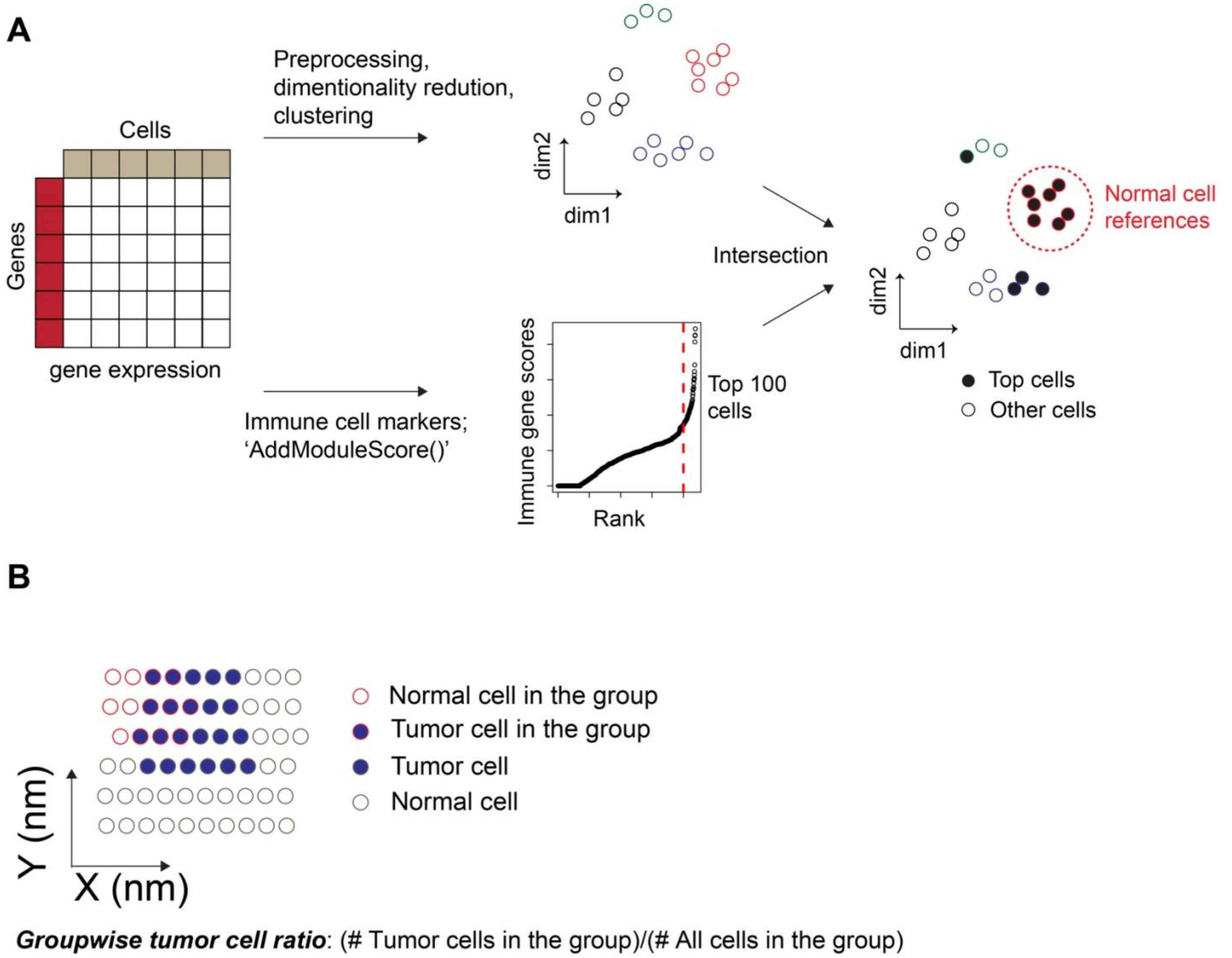

Supplementary Figure 4. Strategy of automatically detection normal cell references and calculate tumor cell ratio in each local group.

A. Strategy to infer immune cell clusters for annotating tumor regions with CopyKat.

B. Calculation of groupwise tumor cell ratio. Red circles represent cells in a local group while grey circles are other cells. Dark blue labels tumor cells. For each group, the tumor ratio equals to the number of tumor cells in this group divided by the number of all cells in the group.

bioRxiv preprint doi: https://doi.org/10.1101/2024.03.19.585725; this version posted March 21, 2024. The copyright holder for this preprint (which was not certified by peer review) is the author/funder, who has granted bioRxiv a license to display the preprint in perpetuity. It is made available under aCC-BY 4.0 International license.

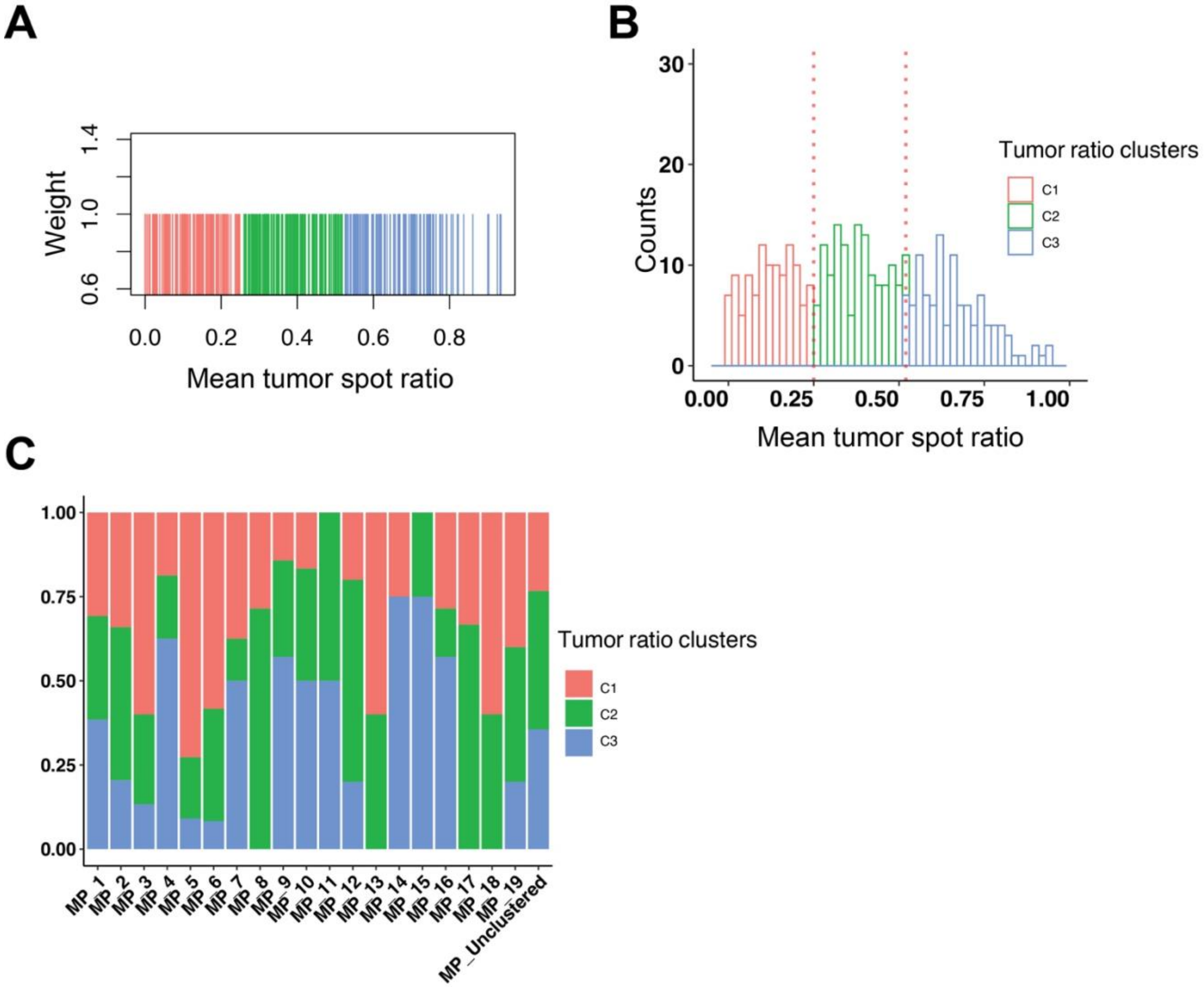

Supplementary Figure 5. Cluster programs based on tumor spot ratios.

A. Using one-dimensional equal-weighted KNN clustering of tumor cell ratios to form three TRCs.

B. The distribution of tumor cell ratios among programs (right). Colors indicate the identity of three TRCs.

C. Proportion of programs clustered to the three TRCs in each meta-program and the ‘unclustered’ programs.

bioRxiv preprint doi: https://doi.org/10.1101/2024.03.19.585725; this version posted March 21, 2024. The copyright holder for this preprint (which was not certified by peer review) is the author/funder, who has granted bioRxiv a license to display the preprint in perpetuity. It is made available under aCC-BY 4.0 International license.

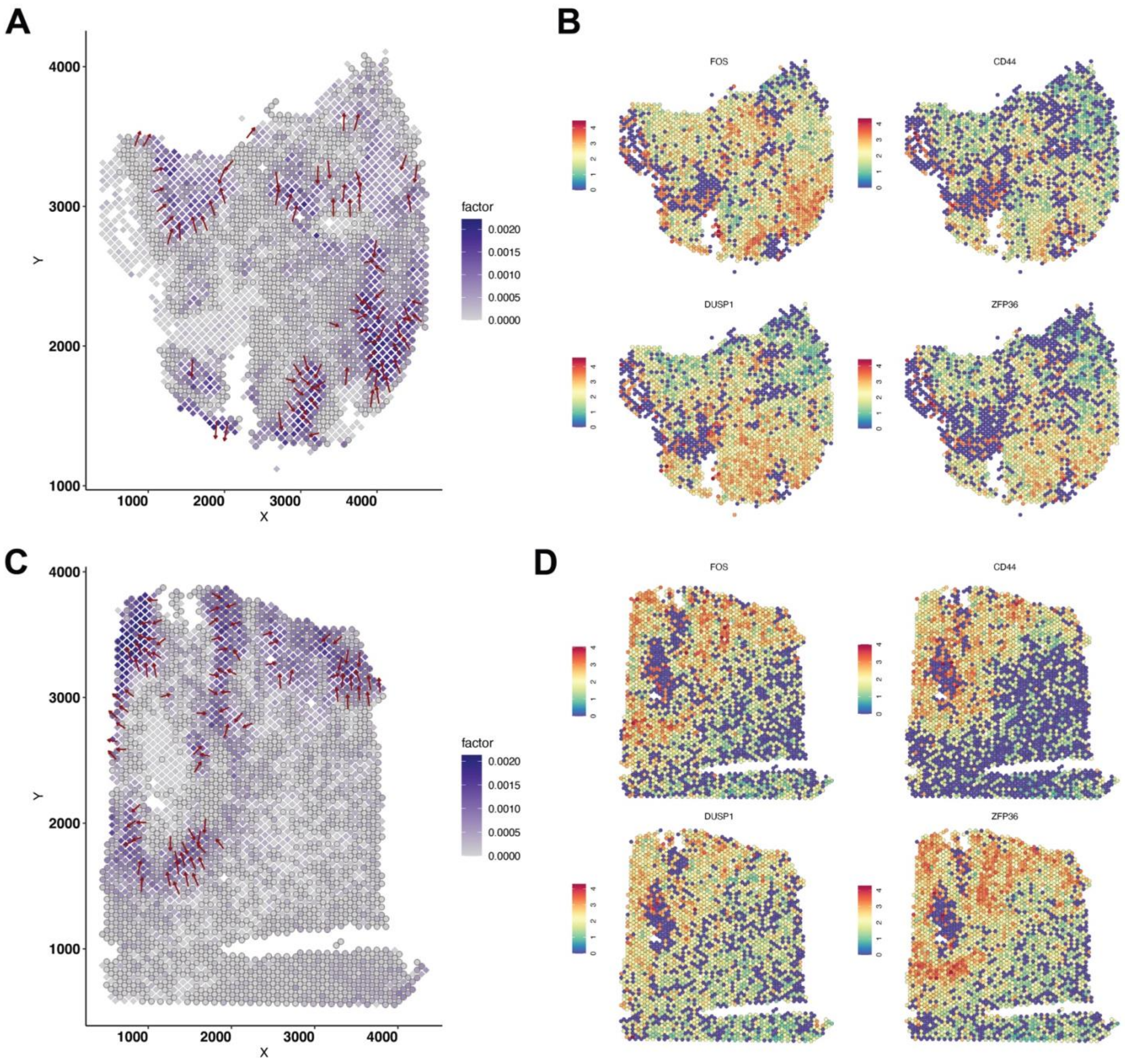

Supplementary Figure 6.

A. The gradient direction and original cell loadings of NMF_3 (UKF55_T_ST) on the spatial map. The overlaying dark grey circles represent data spots characterized as tumor region.

B. The spatial expression of representative genes in NMF_3 (UKF255_T_ST). Warmer colors (red) indicate higher expression levels.

C. The gradient direction and original cell loadings of NMF_6 (UKF260_T_ST) on the spatial map. The overlaying dark grey circles represent data spots characterized as tumor region.

D. The spatial expression of representative genes in NMF_6 (UKF260_T_ST). Warmer colors (red) indicate higher expression levels.
